# Accelerated cardiac magnetic resonance imaging using deep learning for volumetric assessment in children

**DOI:** 10.1007/s00247-024-05978-6

**Published:** 2024-07-17

**Authors:** Melina Koechli, Fraser M. Callaghan, Barbara E. U. Burkhardt, Maélène Lohézic, Xucheng Zhu, Beate Rücker, Emanuela R. Valsangiacomo Buechel, Christian J. Kellenberger, Julia Geiger

**Affiliations:** 1 Department of Diagnostic Imaging, University Children’s Hospital Zurich, Zurich, Switzerland; 2 Children’s Research Center, University Children’s Hospital Zurich, Zurich, Switzerland; 3 Center for MR-Research, University Children’s Hospital Zurich, Zurich, Switzerland; 4 Pediatric Heart Center, University Children’s Hospital Zurich, Zurich, Switzerland; 5 GE HealthCare, Zurich, Switzerland; 6 GE HealthCare, Menlo Park, CA USA

**Keywords:** Cardiac imaging, Deep learning, Image quality, Magnetic resonance imaging, Pediatrics, Ventricular volumetry

## Abstract

**Background:**

Ventricular volumetry using a short-axis stack of two-dimensional (D) cine balanced steady-state free precession (bSSFP) sequences is crucial in any cardiac magnetic resonance imaging (MRI) examination. This task becomes particularly challenging in children due to multiple breath-holds.

**Objective:**

To assess the diagnostic performance of accelerated 3-RR cine MRI sequences using deep learning reconstruction compared with standard 2-D cine bSSFP sequences.

**Material and methods:**

Twenty-nine consecutive patients (mean age 11 ± 5, median 12, range 1–17 years) undergoing cardiac MRI were scanned with a conventional segmented 2-D cine and a deep learning accelerated cine (three heartbeats) acquisition on a 1.5-tesla scanner. Short-axis volumetrics were performed (semi-)automatically in both datasets retrospectively by two experienced readers who visually assessed image quality employing a 4-point grading scale. Scan times and image quality were compared using the Wilcoxon rank-sum test. Volumetrics were assessed with linear regression and Bland–Altman analyses, and measurement agreement with intraclass correlation coefficient (ICC).

**Results:**

Mean acquisition time was significantly reduced with the 3-RR deep learning cine compared to the standard cine sequence (45.5 ± 13.8 s vs. 218.3 ± 44.8 s; *P* < 0.001). No significant differences in biventricular volumetrics were found. Left ventricular (LV) mass was increased in the deep learning cine compared with the standard cine sequence (71.4 ± 33.1 g vs. 69.9 ± 32.5 g; *P* < 0.05). All volumetric measurements had an excellent agreement with ICC > 0.9 except for ejection fraction (EF) (LVEF 0.81, RVEF 0.73). The image quality of deep learning cine images was decreased for end-diastolic and end-systolic contours, papillary muscles, and valve depiction (2.9 ± 0.5 vs. 3.5 ± 0.4; *P* < 0.05).

**Conclusion:**

Deep learning cine volumetrics did not differ significantly from standard cine results except for LV mass, which was slightly overestimated with deep learning cine. Deep learning cine sequences result in a significant reduction in scan time with only slightly lower image quality.

**Graphical Abstract:**

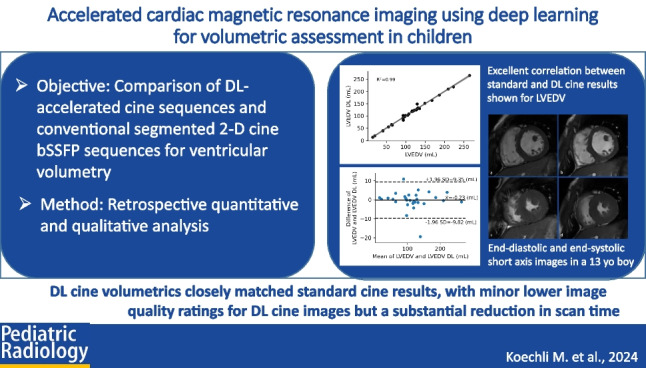

**Supplementary Information:**

The online version contains supplementary material available at 10.1007/s00247-024-05978-6.

## Introduction

Cardiac magnetic resonance imaging (MRI) is the reference standard for quantifying ventricular volumes and function because it is recognized as a more accurate and reproducible method of quantifying ventricular volumes and mass compared to echocardiography [1]. The cine balanced steady-state free precession (bSSFP) sequence is widely employed for evaluating ventricular dimensions (end-diastolic and end-systolic volumes), global function (ejection fraction (EF)), and regional function (wall motion) owing to its inherent blood-to-myocardium contrast and high signal-to-noise ratio [2, 3]. Pediatric normal values for the bSSFP sequence are available [4, 5].

Repetitive breath-holds are necessary during the acquisition of the short-axis stack of two-dimensional (D) cine bSSFP sequences to obtain precise ventricular volumes based on the Simpson method [6]. Ensuring sufficient patient cooperation is crucial to achieve regular and timely consistent breath-holds, thereby obtaining reliable quantitative results [3]. The requirement for intervals of rest between breath-holds makes multiple breath-holds a time-consuming process (typically around 5 min for complete short-axis coverage). Even with cooperative children, performing repetitive breath-holding for up to 20 s can be challenging, leading to artifacts that constrain both image quality and quantitative assessment. Similarly, examinations under anesthesia for younger children require longer rest periods between acquisitions during apnea. Alternatively, free-breathing cine bSSFP acquisitions with multiple signal averages can be performed that generally prolong the examinations.

In recent years, various MRI acceleration methods have been introduced to speed up image acquisition with electrocardiogram-gated bSSFP cine sequences. These technical strategies allow, for instance, free-breathing image acquisition [7–9] or scans with reduced breath-holding duration [10, 11] in pediatric cases. Depending on sequence and scanner properties, techniques such as real-time MRI [8], deep learning–based reconstruction methods of undersampled *k*-space data [7], or acceleration techniques using other spatial and temporal undersampling approaches [10–14] demonstrated promising results.

Recently, a deep learning cine technique was developed that uses a variable density *k*-*t* random sampling scheme and unrolled neural network-based reconstruction. Deep learning cine enables much higher acceleration compared to conventional 2-D bSSFP cine acquisition with retrospective cardiac gating. A similar deep learning approach has already been applied in an adult patient cohort [9] and in children for free-breathing real-time cine acquisition [7]. According to these studies, free-breathing real-time deep learning cine bSSFP shows comparable global cardiac function measurements compared to conventional cine; however, the image quality was relatively compromised when using deep learning cine under the real-time model. Combining multiple-heartbeat (R-R) acquisition with deep learning cine may have a better trade-off between scan time and image quality for patients who can hold their breath.

The aim of this study was to evaluate the quantitative and qualitative performance of the accelerated deep learning cine using the three-heartbeat (3-RR) acquisition protocol compared to the standard 2-D cine bSSFP sequence. The evaluation was conducted in a clinical setting involving children with various types of congenital or acquired heart disease.

We hypothesized that the cine deep learning bSSFP sequence provided similar ventricular volumes, function, and image quality with a significantly shorter scan time.

## Material and methods

### Patient population

Thirty consecutive patients who underwent cardiac MRI for clinical reasons between June 2022 and February 2023 were identified for this retrospective study. The study was approved by the local ethics committee (BASEC no. 2023–00703). Written informed consent was obtained from the children’s parents or legal guardians before the examination.

### Magnetic resonance imaging

All patients were examined on a 1.5-tesla scanner (Signa Artist, GE Healthcare, Chicago, IL) using an anterior and posterior 20-channel AIR™ coil (GE Healthcare). All children were scanned with a conventional breath-hold 2-D bSSFP cine sequence and an accelerated deep learning cine 3-RR sequence in the short axis (sequences for each child were performed in random order). The deep learning cine sequence enables the acquisition of 2-D cine bSSFP sequences using one to six heartbeats per slice with high acceleration based on variable density *k*-*t* random sampling and deep learning reconstruction.

Both sequences were acquired using retrospective electrocardiogram gating with comparable parameters concerning slice thickness, matrix, echo, and repetition times. The slice thickness was 8 mm with a 2-mm gap but was reduced to 5–7 mm without a gap in order to obtain a reasonable number of slices for ventricular volume assessment in five younger and smaller patients (in the age range of 1–5 years). For coverage of the entire heart, 12 slices were acquired in 20, 13–14 slices in eight, and 11 slices in two patients. In addition, left ventricular (LV) two-chamber and four-chamber views were acquired for both sequences in 22 patients. Multiple breath-holds (each with 15–18 s) were performed according to the number of slices for the acquisition of the conventional 2-D cine sequence. Using the deep learning cine 3-RR sequence, three to four slices were acquired within one breath-hold (lasting 12–16 s), resulting in three to four breath-holds for a complete short-axis stack acquisition. For those seven patients who were scanned under general anesthesia, the breath-holds were performed with apnea. The deep learning cine images were reconstructed on the scanner within 10–15 min without impairing the ongoing scanning.

For conventional 2-D cine, six to eight views per segment according to the heart rate were used, targeting a temporal resolution of about 20–30 ms (i.e., 30–40 cardiac phases/heartbeat). The deep learning cine sequence used ten views per segment with a reconstructed temporal resolution in the same range.

Sequence parameters are shown in Table 1.

**Table 1:** Imaging parameters for conventional cine and deep learning cine sequences

Sequence parameters	Conventional cine	Deep learning cine
ECG gating	Retrospective	Retrospective
TR/TE (ms)	3.62–4.20/1.66–1.94	3.28–3.94/1.13–1.69
Flip angle (°)	45	49–55
Acquired spatial resolution (mm^2^)	1.3–1.8	1.3–1.8
Reconstructed spatial resolution (mm^2^)	0.5–0.7 × 0.5–0.7	0.5–0.7 × 0.5–0.7
Acquired temporal resolution (ms)	16–31	17–33
Slice thickness (mm)	5–8	5–8
Slice gap (mm)	0–2	0–2
Matrix	160–224 × 200–224	200 × 200
Number of slices	12 (11–14)	12 (11–14)
Breath-holds	12 (11–14)	3–4

*ECG* electrocardiogram, *TE* echo time, *TR* repetition time

### Quantitative analysis

All images were assessed using cloud-based commercially available postprocessing software (Tempus Pixel, Chicago, IL). Images derived from the short axis were segmented (semi-)manually in end-diastole and end-systole. This involved adapting the automatically generated endocardial biventricular contours for volume measurements and LV epicardial contours for quantifying LV mass. One pediatric radiologist (J.G. with 15 years of experience in cardiovascular MRI) and one third-year medical student (M.K.) performed the quantitative volumetric postprocessing independently. Left ventricular end-diastolic volumes (LVEDV), left ventricular end-systolic volumes (LVESV), left ventricular stroke volumes (LVSV), LV ejection fraction (LVEF), LV mass, right ventricular end-diastolic volumes (RVEDV), RV end-systolic volumes (RVESV), RV stroke volumes (RVSV), and RV ejection fraction (RVEF) as well as volumes normalized to body surface area were recorded for both the conventional and deep learning cine sequences, respectively. The papillary muscles were included in the blood pool and therefore not part of the LV mass.

### Qualitative image analysis

Two experienced pediatric cardiovascular physicians (one radiologist (J.G.) and one cardiologist (B.E.U.B., with 15 years of experience in cardiovascular MRI), blinded to the participant demographics and sequence type, independently scored image quality of the standard and deep learning cine images. Image quality scores were based on four criteria: end-diastolic and end-systolic contour delineation, visibility of papillary muscles and valves. Images in the short-axis stack of both sequences were rated using a 4-point grading system from 1 to 4: 1 = very poor, almost non-diagnostic image quality and structure delineation; 2 = poor image quality/structure delineation; 3 = good image quality/delineation; 4 = excellent image quality/delineation. If LV two-chamber or four-chamber images were available for both sequences, they were included for the assessment of atrioventricular valve depiction. Aortic valves were rated in the short-axis images.

In addition, a 3-D “volumetric mesh” was assessed for both sequences using the same 4-point score as described above. Alongside evaluating image quality, the degree of distortion of the cardiac surface obtained from the postprocessing software was also assessed. The postprocessing software automatically generated this mesh using artificial intelligence algorithms, relying on the end-diastolic contours from the short-axis stack data.

### Statistical analysis

GraphPad Prism (GraphPad Prism version 10, Boston, MA) and Python (Python version 3.8, DE) software were used for all statistical tests. The Shapiro–Wilk test was applied to test normal distribution. Paired *t*-tests were performed to compare ventricular volumes and mass between standard and deep learning cine sequences. Non-paired *t*-tests were performed to compare the results of the congenital heart disease and the non-congenital heart disease groups. Linear regression analysis was applied for the assessment of cardiac function parameters for both sequences. The Bland–Altman plots were created to evaluate the agreement between LV and RV function parameters obtained with standard and deep learning cine sequences.

Differences in scan times and image quality scores were compared with the Wilcoxon rank-sum test. The Spearman correlation coefficient was used to test relationships between image quality, age, and heart rate. The interobserver agreement was assessed using the intraclass correlation coefficient (ICC), correlated to agreement as poor (< 0.5), fair (0.5–0.75), good (0.75–0.9), and excellent (> 0.9) [15]. A significance level of *P* < 0.05 was considered statistically significant.

## Results

### Patients

Thirty patients (mean age 11 ± 5.1 years, median 12 years, interquartile range 9, range 1–17 years) were examined with both sequences. One patient had to be excluded from the study due to cardiomyopathy characterized by severe myocardial hypertrophy that hindered proper ventricular assessment. Seven patients were examined in general anesthesia (mean age 3.7 ± 2.8 years, median 4 years, range 1–9 years).

Patients had various types of congenital heart or aortic disease, with some having undergone prior surgical correction. Two patients were examined to rule out clinically suspected cardiomyopathy. Patients’ heart rate ranged between 50 and 120 bpm (mean 73 ± 20 bpm).

Patient demographics are shown in Tables 1 and 2.

**Table 2:** Patient characteristics

**Demographics**	Total *n*=29
Sex	18 male/11 female
Age (years)	11 ± 5.1
Height (cm)	148.4 ± 33.3
Weight (kg)	44.8 ± 21.6
BSA (m^2^)	1.4 ± 0.5
***Diagnoses***	
***Aortic pathologies***	
Aortic coarctation	6 (*n*=3 with BAV)
Double aortic arch, LVNC	1
Marfan syndrome	4
Right aortic arch, S/p resection of diverticulum of Kommerell	1
***Valve disease or congenital heart disease***	
Mitral valve prolapse	2
PAPVC/sinus venosus ASD	4
DTGA after arterial switch operation	2
Coronary anomaly	1
S/p surgery for RVOT obstruction	1
S/p correction for TAC	2
S/p pulmonary atresia surgery	1
S/p VSD closure	1
S/p Ross procedure	1
***Acquired heart disease***	2
Exclusion of suspected cardiomyopathy	

*ASD* atrial septal defect, *BAV* bicuspid aortic valve, *BSA* body surface area, *DTGA* dextro-transposition of the great arteries, *LVNC* left ventricular non-compaction, *PAPVC* partial anomalous pulmonary venous connection, *S/p* status post, *RVOT* right ventricular outflow tract, *TAC* common arterial trunk, *VSD* ventricular septal defect

### Scan times

The mean scan time for a short-axis stack acquisition was 218.3 ± 44.8 s in the standard 2-D cine sequence and 45.5 ± 13.8 s for the 3-RR deep learning cine sequence, resulting in a significant reduction (80%) of mean data acquisition time (*P* < 0.001). In conventional cine acquisition, 12 (range 11–14) breath-holds were performed to acquire a complete short-axis stack, whereas only three breath-holds were needed for the 3-RR deep learning cine sequence.

### Quantitative results

#### Left ventricle

We did not detect significant differences between the two sequences for left ventricular (LV) volumetric results except for LV mass (Table 3). LVEDV was 116.3 ± 56.4 ml in standard cine vs. 116.5 ± 56.7 ml in deep learning cine (*P* = 0.8), LVESV 48.7 ± 23.4 ml vs. 48.8 ± 23.4 ml (*P* = 0.9), LVSV 67.6 ± 33.9 ml vs. 67.9 ± 33.9 ml (*P* = 0.8), and LVEF 58 ± 4.3% vs. 58 ± 4.1% (*P* = 0.9). LV mass was significantly increased for deep learning cine (71.4 ± 33.1 g) compared to standard cine (70 ± 32.5 g; *P* = 0.008).

**Table 3:** Volumetric results

	**Conv cine**	**DL cine**	***P*-value**^**a**^	**ICC (Conv/DL cine)**
LVEDV (ml)	116.3 ± 56.4	116.5 ± 56.7	0.80	1.00/1.00
LVESV (ml)	48.7 ± 23.4	48.8 ± 23.4	0.92	0.98/0.98
LVSV (ml)	67.6 ± 33.9	67.8 ± 33.9	0.82	0.99/0.96
LVEF (%)	58.0 ± 4.3	58.0 ± 4.1	0.99	0.81/0.81
LV mass (g)	69.9 ± 32.5	71.4 ± 33.1	**0.008**	0.98/0.97
RVEDV (ml)	128.3 ± 57.4	127.5 ± 58.7	0.22	0.99/0.99
RVESV (ml)	59.9 ± 27.5	59.3 ± 28.0	0.32	0.96/0.97
RVSV (ml)	68.4 ± 32.0	68.3 ± 32.7	0.75	0.96/0.98
RVEF (%)	53.4 ± 5.9	53.4 ± 6	0.84	0.73/0.81

*Conv* conventional, *DL* deep learning, *ICC* intraclass coefficient, *LV mass* left ventricular mass, *LVEDV* left ventricular end-diastolic volume, *LVEF* left ventricular ejection fraction, *LVESV* left ventricular end-systolic volume, *LVSV* left ventricular stroke volume, *RVEDV* right ventricular end-diastolic volume, *RVEF* right ventricular ejection fraction, *RVESV* right ventricular end-systolic volume, *RVSV* right ventricular stroke volume

^a^Bold represents statistical significance (*P* < 0.05)

Normalized values to body surface area did not differ between the two sequences (LVEDV conventional cine 84.1 ± 24.2 ml/m^2^ vs. deep learning cine 83.9 ± 23.6 ml/m^2^). Seven patients had LV dilatation (*Z* > 2). When dividing the patients into two subgroups, one with congenital heart disease and the other without congenital heart disease, we also did not observe any significant differences between the two sequences. Results of the congenital heart disease group are summarized in Table 4.

**Table 4:** Volumetrics in the congenital heart disease group

	**Conv cine**	**DL cine**	***P*-value**^**a**^
LVEDV (ml)	87.9 ± 40.9	87.4 ± 41.2	0.64
LVESV (ml)	37.5 ± 17.6	37.6 ± 17.5	0.80
LVSV (ml)	50.4 ± 24.4	49.7 ± 24.3	0.56
LVEF (%)	57.5 ± 4.9	57.2 ± 4.5	0.55
LV mass (g)	53.5 ± 25.0	54.8 ± 24.9	0.07
RVEDV (ml)	108.6 ± 52.3	106.8 ± 52.9	**0.03**
RVESV (ml)	51.6 ± 25.4	50.7 ± 26.7	0.36
RVSV (ml)	57.0 ± 28.3	56.0 ± 27.7	0.17
RVEF (%)	53.2 ± 5.7	53.3 ± 5.8	0.85

*Conv* conventional, *DL* deep learning, *LV mass* left ventricular mass, *LVEDV* left ventricular end-diastolic volume, *LVEF* left ventricular ejection fraction, *LVESV* left ventricular end-systolic volume, *LVSV* left ventricular stroke volume, *RVEDV* right ventricular end-diastolic volume, *RVEF* right ventricular ejection fraction, *RVESV* right ventricular end-systolic volume, *RVSV* right ventricular stroke volume

^a^Bold represents statistical significance (*P* < 0.05)

**Fig. 1:**
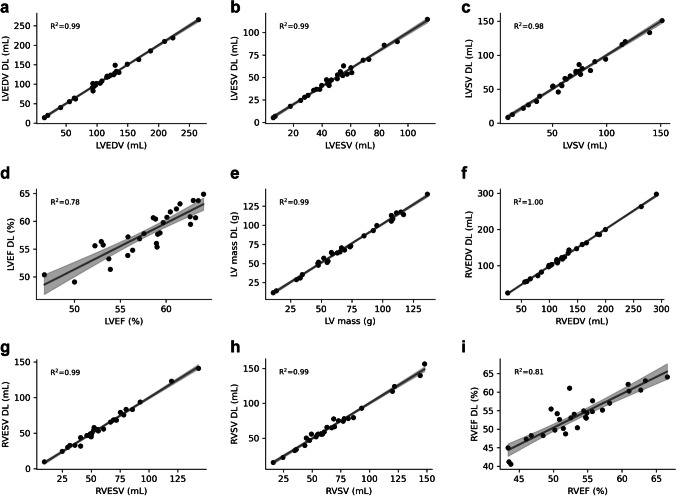
Linear regression analyses for LV (**a**-**e**) and RV (**f**-**i**) volumetric measurements in standard cine and deep learning cine demonstrate excellent correlations for EDV (**a**, **f**), ESV (**b**, **g**), SV (**c**, **h**), EF (**d**, **i**) and mass (**e**). The regression lines and coefficients of determination are integrated into the graphs. *DL* deep learning, *EDV* end-diastolic volume, *ESV* end-systolic volume, *EF* ejection fraction, *LV* left ventricular, *RV* right ventricular, *SV* stroke volume

**Fig. 2:**
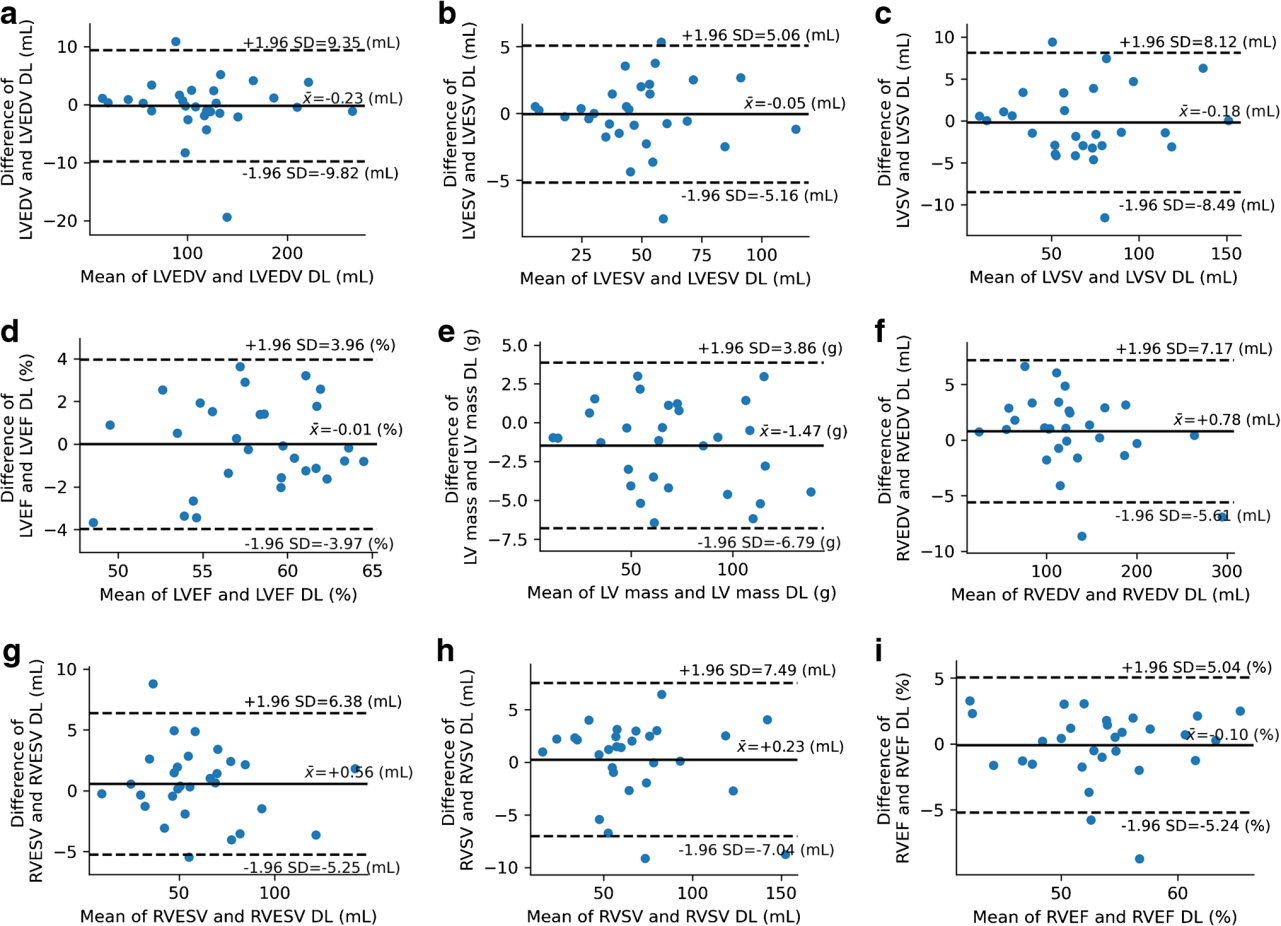
Correlation between standard cine and deep learning cine for LV and RV volumes and function. Bland–Altman plots for LV (**a**-**e**) and RV (**f**-**i**) show the calculated difference between standard cine and deep learning cine sequences on the *y*-axis as a function of the mean of both sequences on the *x*-axis for EDV (**a**, **f**), ESV (**b**, **g**), SV (**c**, **h**), EF (**d**, **i**) and mass (**e**). Solid lines represent the mean value of the differences (standard cine − DL cine); broken lines demonstrate the mean difference ± 1.96 SD. *DL* deep learning, *EDV* end-diastolic volume, *ESV* end-systolic volume, *EF* ejection fraction, *LV* left ventricular, *RV* right ventricular, *SD* standard deviation, *SV* stroke volume

Linear regression revealed good correlations between the two sequences for all parameters (Fig. 1). The Bland–Altman analysis demonstrated low bias and small limits of agreement between the conventional and deep learning cine results (LVEDV -0.2 ± 4.9 ml, LVESV 0 ± 2.6 ml, LVSV -0.2 ± 4.3 ml, LVEF 0 ± 2.1%, LV mass -1.5 ± 2.8 g) (Fig. 2).

#### Right ventricle

Right ventricular (RV) volumetrics did not reveal significant differences between the two methods (Table 3). Similar to LV, close correlations between conventional and deep learning cine results were found in linear regression analysis (Fig. 1). RVEDV (128.3 ± 57.4 ml) and RVESV (59.9 ± 27.5) were slightly, but not significantly, increased in conventional cine compared with deep learning cine images (RVEDV 127.5 ± 58.7 ml; *P* = 0.2/RVESV 59.3 ± 28 ml; *P* = 0.3). RVSV was not significantly increased for standard cine compared with deep learning cine results (68.4 ± 32.0 ml vs. 68.2 ± 32.7 ml; *P* = 0.7). RVEDV indexed to body surface area revealed a significant difference between the two sequences (conventional cine 96.4 ± 25.9 ml/m^2^ vs. deep learning cine 95 ± 25.5 ml/m^2^; *P* = 0.03). Six of twenty-nine patients had an enlarged right ventricle (*Z* > 2), 5/15 in the congenital heart disease group. RV results did not differ between the two sequences for patients with and without congenital heart disease except for RVEDV (conventional cine 108.5 ± 52.3 ml vs. deep learning cine 106.8 ± 52.9 ml; *P* = 0.03) (Table 4).

The Bland–Altman plots revealed a more pronounced bias for RV volumes compared to LV volumes and narrow limits of agreement (RVEDV 0.7 ± 3.3 ml, RVESV 0.6 ± 3.0 ml, RVSV 0.3 ± 3.8 ml, RVEF -0.1 ± 2.7%) (Fig. 2).

#### Reproducibility

The interobserver agreement was excellent between most left and right volumetric measurements and LV mass derived from standard cine and deep learning cine images (ICC 0.98–1). We observed a good correlation for LVEF for both sequence types (ICC 0.81) and RVEF for deep learning cine (ICC 0.81), and an almost good correlation for RVEF using standard cine images (ICC 0.73). All values are summarized in Table 3.

### Qualitative results

The mean overall image quality score for conventional 2-D cine images was significantly higher including depiction of end-diastolic (ED) and end-systolic (ES) contours, papillary muscles, and valve visualization compared with deep learning cine images (3.5 ± 0.4 vs. 2.9 ± 0.5; *P* < 0.001). Deep learning cine image quality was rated more than one point lower for ES contours, papillary muscles, and valves for deep learning cine sequences as compared to standard cine images by at least one reader.

The mean image score for ED contour delineation in standard 2-D cine images was 3.8 ± 0.4 compared to 3.4 ± 0.6 in deep learning cine images (*P* = 0.04). The difference was more pronounced for ES contours revealing a mean score of 3.6 ± 0.5 for conventional cine images compared with 2.8 ± 0.6 for deep learning cine images (*P* < 0.001). A similar observation was found comparing papillary muscles (standard cine 3.6 ± 0.6 vs. deep learning cine 3.0 ± 0.8; *P* < 0.001) and valve depiction (standard cine 3.3 ± 0.6; deep learning cine 2.0 ± 0.7; *P* < 0.001). Mesh surface quality was rated slightly higher for deep learning cine (3.1 ± 0.8) compared with conventional cine sequences (3.1 ± 0.7; *P* = 0.8). The discrepancy in image quality was independent from sedation in younger children. We did not observe an age dependency for image quality. There was no relationship between heart rate and image quality for either sequence.

**Fig. 3:**
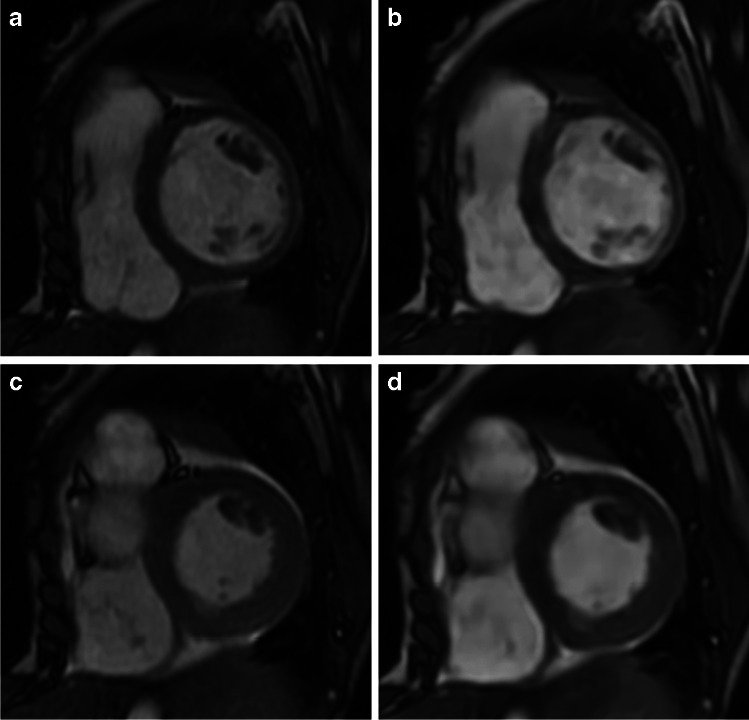
Example images of different image quality between conventional 2-dimensional cine and deep learning cine images in a 2-year-old boy with Marfan syndrome who was examined under general anesthesia. Images in short-axis orientation in a basal slice during end-diastole (**a**, **b**) and end-systole (**c**, **d**). **a** and **c** were acquired with the conventional cine sequence revealing a mean rating of 4 for end-diastolic (ED) contour, 3.5 for end-systolic (ES) contour, and 3.5 for papillary muscles (PM). **b** and **d** are deep learning cine images at the same slice position having mean scores of 3 (ED), 2 (ES), and 2 (PM)

**Fig. 4:**
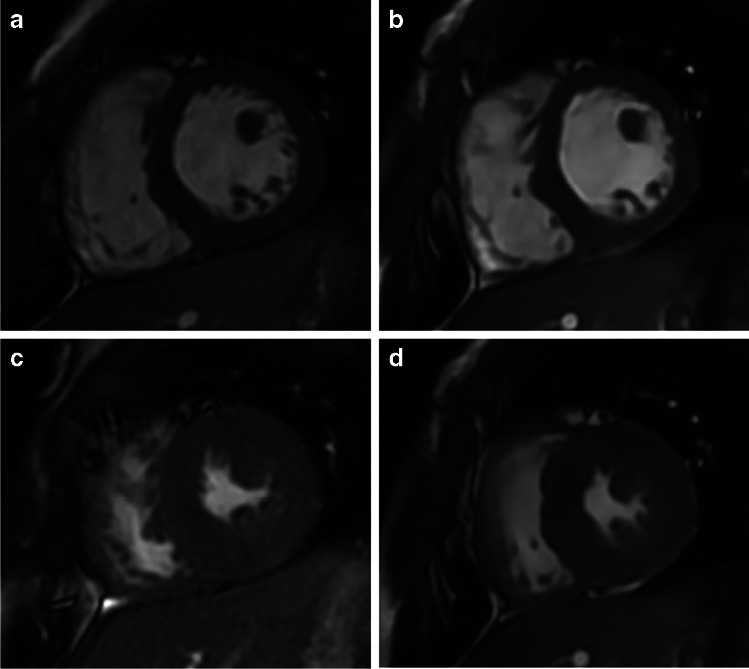
Example high-quality images of conventional 2-dimensional cine (**a**, **c**) and deep learning cine images (**b**, **d**) in a 13-year-old boy after vascular ring repair. Images in a midventricular position of the short axis during end-diastole (**a**, **b**) and end-systole (**c**, **d**). Mean image scores of the conventional image were 4 for end-diastolic (ED) contours, 4 for end-systolic (ES) contours, and 4 for papillary muscles (PM), whereas deep learning cine images were rated with 4 (ED), 3.5 (ES), and 4 (PM)

**Fig. 5:**
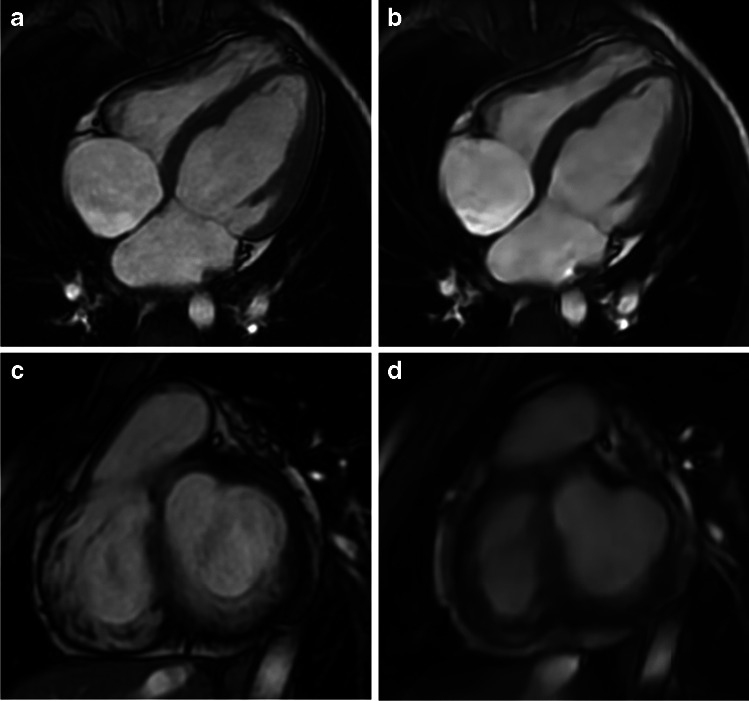
Example case of a 9-year-old boy after correction of an aberrant left coronary artery. Images in 4-chamber view (**a**, **b**) and short axis (**c**, **d**) of conventional cine (**a**, **c**) and deep learning cine (**b**, **d**) images for valve depiction. Mean rating of the conventional images for valve delineation was 4 compared to 2 in the deep learning cine images

**Fig. 6:**
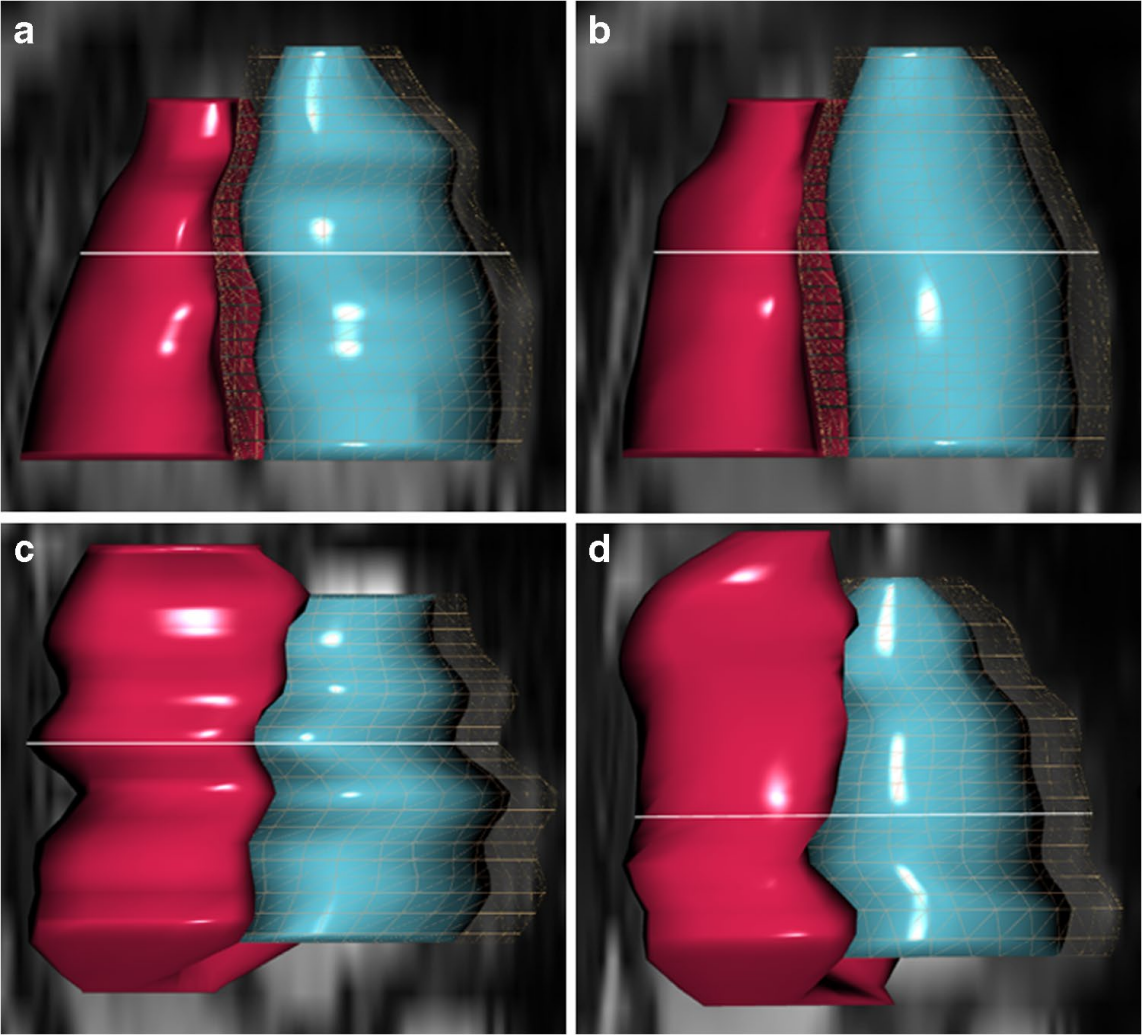
Auto-generated 3-dimensional mesh surface contours based on conventional and deep learning cine images of two patients as representative examples of good and poor image quality. The right ventricle is shown in red and the left ventricle in turquoise. **a**, **b** A 16-year-old adolescent boy after surgical correction for partial anomalous venous drainage. Conventional cine (**a**) had a lower score of 3 compared to the score of 4 for deep learning cine mesh. **c**, **d** A 9-year-old boy after coronavirus infection showing poor quality for both sequences (**c** conventional cine, **d** deep learning cine) with mean scores of 1 and 2, respectively, as a result of distortions due to inconsistent breathing

Examples of conventional and deep learning cine images at ED and ES are shown in Figs. 3 and 4 as well as in the two videos in Online Supplementary Materials 1 and 2. Figure 5 shows a representative example for valve depiction including a four-chamber view. Figure 6 depicts examples of high and poor mesh surface quality for both sequences.

## Discussion

In this study, we performed clinical validation of an accelerated deep learning cine bSSFP sequence (3-RR interval acquisition) in comparison to a standard 2-D cine bSSFP sequence in 29 children with various cardiovascular diseases. We found that the deep learning cine sequence allowed a significant reduction in acquisition time with only slightly lower image quality. We did not find any significant differences in biventricular volumes and EF between the two sequences.

The standard approach for volumetric and functional assessment in cardiac MRI involves acquiring a short-axis stack of 2-D bSSFP cine slices. However, a notable limitation of this conventional technique is the requirement for multiple breath-holds to mitigate respiratory motion artifacts. This proves particularly challenging for critically ill patients and younger children who may find it difficult to remain still during the procedure. Arrhythmia can also give rise to technical issues and impaired image quality due to the retrospective electrocardiogram gating employed in the conventional segmented cine bSSFP sequence. Typically, segments of the entire *k*-space are read out over multiple cardiac cycles in this cine sequence type using several RR intervals.

The application of free-breathing sequences without the need for breath-holds or accelerated image acquisitions using less RR intervals may overcome these important limitations. In recent years, several techniques based on deep learning have been developed to achieve significant advancements in radiological applications. Specifically, the focus has been on accelerating scanning processes, minimizing artifacts, and maintaining image quality in cardiac MRI [16–19].

Our study applied a newly developed deep learning cine sequence with variable density *k*-*t* random sampling and deep learning reconstruction. This sequence uses retrospective cardiac gating and enables accelerated 2-D cine bSSFP image acquisition based on one to six heartbeats. In this study, we applied the 3-RR deep learning cine version, which means that the images were acquired based on three heartbeats. Thus, image acquisition was considerably shorter (80%) compared with the standard cine sequence. A reduced number of breath-holds (three instead of 12) were necessary for acquiring a short-axis stack, as multiple slices could be obtained within a single breath-hold.

LV and RV volumetrics revealed a strong correlation between the deep learning cine and the standard cine sequences with narrow limits of agreement. Interobserver agreement was excellent even in a clinical setting with several patients with complex congenital heart disease. Challenges may arise in accurately defining endocardial contours due to abnormal anatomy and frequently enlarged right ventricles in patients with congenital heart disease [20]. This may be the reason for the slight underestimation of RVEDV in the deep learning cine sequences that we observed in the patients with congenital heart disease. Moreover, we obtained excellent reproducibility of the measurements, despite the fact that the second reader was a third-year medical student without prior experience in cardiovascular MRI.

We assume that a minor difference in the epicardial contours may be the origin of the statistically significant but not clinically relevant difference in LV mass overestimation in the deep learning cine sequence.

A similar methodological approach was described in a recent study by Orii et al. using a respiratory-gated real-time cine sequence with deep learning reconstruction for volumetric analysis in adults [9]. Similar to our results, they did not observe any significant differences in volumetrics between the deep learning reconstruction cine and the conventional 2-D cine sequence and reported shorter scan times for the deep learning reconstruction cine sequence. In contrast, they report an underestimation of LV mass in the deep learning reconstruction cine, potentially attributed to technical limitations leading to blurred images.

Another recent study applied a highly accelerated, model-based, free-breathing deep learning method in children and young adults [7]. The comparison of this model-based reconstruction method of undersampled *k*-space data [21] with the standard 2-D bSSFP cine sequences demonstrated excellent agreement for biventricular volumes and LV mass and a considerably shorter acquisition time using the deep learning sequence.

In the past, real-time imaging for facilitating volumetric image acquisition in adults was investigated by different groups [22–26]. A recent publication by Röwer et al. investigated the performance of a real-time MRI sequence with free-breathing in children in comparison to a standard breath-hold acquisition, resulting in comparable volumetric and functional results [8]. Although real-time imaging during free breathing seems very favorable for children with regard to patient compliance and physiological cardiac and pulmonary conditions, this technique requires dedicated postprocessing software and is not yet widely available.

Instead of free-breathing real-time imaging, compressed sensing techniques allow for fast scan times and shorter breath-holds [10–14, 27, 28]. In a recent study by Naresh et al., no significant differences in volumetric assessment were reported when validating a compressed sensing method against the standard cine bSSFP sequence in children and young adults [10]. However, the compressed sensing technique only resulted in a 43% reduction in scan time and a 50% decrease in the number of required breath-holds, while our comparison resulted in 80% shorter acquisition times using deep learning cine.

In our study, the overall image quality received lower ratings from both readers. The overall image score for deep learning cine was 0.6 points lower compared to standard cine images (2.9 ± 0.5 vs. 3.5 ± 0.4), indicating that deep learning cine images achieved an almost good diagnostic quality of 3. We believe that the overall image quality of deep learning cine is sufficient for diagnostic purposes and does not impair therapeutic decision. The difference was quite small for end-diastolic contours (score difference 0.4) and more obvious for end-systolic contours (difference 0.8) and papillary muscles (difference 0.6). Specifically, the identification of the aortic valve in the short axis and the atrioventricular valves in the four-chamber view was significantly compromised in the deep learning cine images with an average score of 2. The use of general anesthesia in young children did not affect the difference in image quality. We also excluded other factors such as age or heart rate which did not reveal relationships with image quality.

In the qualitative analysis conducted by Orii et al., diminished overall image quality was observed in deep learning reconstruction cine images, attributed to inferior endocardial edge definition and an increased presence of motion artifacts [9]. The overall image quality in the real-time MRI study only revealed minor differences between the sequences, with a lower rating observed for papillary muscles and blood pool contrast for real-time images [8]. Qualitative image evaluation did not reveal significant differences with regard to edge definition, blood-myocardial contrast, and artifacts in the study comparing compressed sensing and standard cine bSSFP sequences [10].

This study has several limitations. It is a single-center study with a rather small number of patients. Since we sought to assess both sequences in a clinical setting, our study population presented with a wide age range. Young patients were examined under general anesthesia while the majority were examined without sedation. Factors such as breathing inconsistencies and potential moving artifacts in the older and awake patients may influence image quality.

We performed the study with a standardized scan protocol using only the 3-RR deep learning sequence, which is a compromise between the available 1-RR and 6-RR options. Although it would be interesting to compare different RR intervals, a direct comparison of various RR intervals is not feasible in a clinical routine because this would prolong the examination beyond accepted limits of time. Moreover, the protocol used was not yet completely optimized for pediatric scanning, and further improvement in acceleration factor and spatial and temporal resolutions could be achieved in the future.

The second reader was an inexperienced medical student without prior training in cardiac MRI. Nevertheless, we achieved excellent interobserver agreement in volumetric assessment of both sequences.

The current deep learning cine image reconstruction time on the scanner, which takes up to 15 min on the central processing unit, is expected to be reduced in the future through optimization of the deep learning model. This improvement aims to provide immediate availability of images following the scan.

Further investigation involving a larger number of patients is needed to evaluate the strength of the deep learning cine technique more comprehensively. Moreover, further advancements in sequence development are necessary, focusing on the creation of free-breathing deep learning sequences that maintain high image quality, ultimately eliminating the need for breath-holds or anesthesia with intubation.

## Conclusion

In conclusion, this highly accelerated deep learning cine MRI sequence achieves similar biventricular volume and function results when compared with those of the reference standard cine SSFP sequence. Deep learning cine sequences significantly reduce the scan time for functional cardiac analysis in children. In the pediatric population or in patients with limited compliance or significant arrhythmias, these new sequences represent a major improvement in clinical routine imaging.

## Supplementary Information

Below is the link to the electronic supplementary material.

Supplementary file1 (MPG 1.93 MB)

Supplementary file2 (MPG 2.49 MB)

## Data Availability

The data used and/or analyzed in this study are available from the corresponding author on reasonable request.
